# Bio-Anthropological Studies on Human Skeletons from the 6th Century Tomb of Ancient Silla Kingdom in South Korea

**DOI:** 10.1371/journal.pone.0156632

**Published:** 2016-06-01

**Authors:** Won-Joon Lee, Eun Jin Woo, Chang Seok Oh, Jeong A. Yoo, Yi-Suk Kim, Jong Ha Hong, A. Young Yoon, Caroline M. Wilkinson, Jin Og Ju, Soon Jo Choi, Soong Doek Lee, Dong Hoon Shin

**Affiliations:** 1 Institute of Forensic Science, Seoul National University College of Medicine, Seoul, South Korea; 2 Division in Anatomy & Developmental Biology, Department of Oral Biology, BK21 PLUS Project, Yonsei University College of Dentistry, Seoul, South Korea; 3 Bioanthropology and Paleopathology Lab, Department of Anatomy, Seoul National University College of Medicine, Seoul, South Korea; 4 Department of Anatomy, Ewha Womans University School of Medicine, Seoul, South Korea; 5 Visual Communication Design, Sungkyunkwan University, Seoul, South Korea; 6 Face Lab, Liverpool Science Park IC1, 131 Mount Pleasant, Liverpool John Moores University, Liverpool, United Kingdom; 7 Foundation of Silla Cultural Heritage Research Institute, Gyeongju, South Korea; Hebrew University, ISRAEL

## Abstract

In November and December 2013, unidentified human skeletal remains buried in a *mokgwakmyo* (a traditional wooden coffin) were unearthed while conducting an archaeological investigation near Gyeongju, which was the capital of the Silla Kingdom (57 BCE– 660 CE) of ancient Korea. The human skeletal remains were preserved in relatively intact condition. In an attempt to obtain biological information on the skeleton, physical anthropological, mitochondrial DNA, stable isotope and craniofacial analyses were carried out. The results indicated that the individual was a female from the Silla period, of 155 ± 5 cm height, who died in her late thirties. The maternal lineage belonged to the haplogroup F1b1a, typical for East Asia, and the diet had been more C_3_- (wheat, rice and potatoes) than C_4_-based (maize, millet and other tropical grains). Finally, the face of the individual was reconstructed utilizing the skull (restored from osseous fragments) and three-dimensional computerized modeling system. This study, applying multi-dimensional approaches within an overall bio-anthropological analysis, was the first attempt to collect holistic biological information on human skeletal remains dating to the Silla Kingdom period of ancient Korea.

## Introduction

The Silla Kingdom, beginning its history as a small city-state, prospered during the first millennium CE (57 BCE– 935 CE), becoming a well-organized state that ruled most of the Korean peninsula. Understanding the history of the Silla Kingdom from an archaeological perspective is very important, as it remains highly relevant. In fact, much of present-day Korea’s cultural heritage is rooted in its traditions.

In Silla funeral culture, various types of tombs corresponding to the different social classes were constructed. Among them, wood coffins (*mokgwanmyo*) and stone-mounted wooden chamber tombs (*jeokseok mokgwangmyo*) are representative. In spite of the importance of Silla tombs from the academic perspective, very few bio-anthropological studies have been carried out, due to the typically degenerated condition of the bones contained therein. This is largely the result of the acidic soil and the alternate hot/wet, cold/dry weather patterns, which tend to accelerate the decomposition of a human cadaver [1]. Archaeologists/anthropologists in South Korea therefore have not had enough opportunities to explore intact ancient human bones from the Silla period, except for a few anthropometrical studies [2], cremation and scanning electron microscope (SEM) studies [3], and stable isotope analysis for dietary pattern studies [4]. These previous studies however are limited, as, having applied only a single method rather than utilizing a holistic approach with a variety of analysis tools, they can provide only partial biological information.

The present investigation represents a rare chance to examine intact human remains dating to the Silla period. Researchers from varied fields collaborated to acquire biological data based on physical anthropological, mitochondrial DNA (mtDNA), stable isotope and craniofacial analysis. This study can provide significant information on the ancient Silla people, which is very meaningful not only to academics but also to the general Korean public.

## Materials and Methods

### Archaeological Information

**Fig 1:**
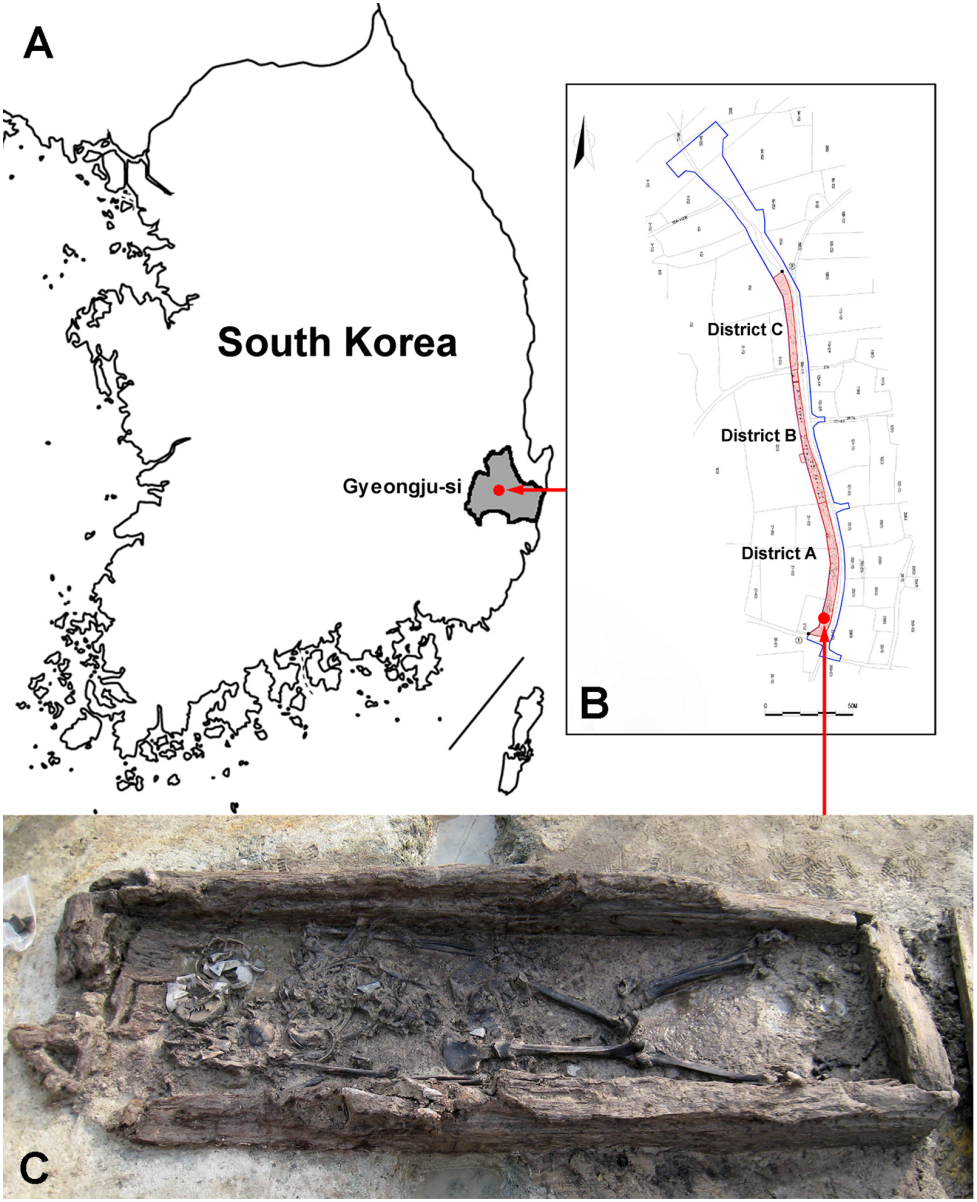
Location of the excavation site and the uncovered coffin. A: the red dot and arrow indicate the location of archaeological excavation site in Gyeongju-si in South Korea; B: the red dot and arrow indicate the location of the wooden coffin (*mokgwanmyo*) at the excavation site; C: the human skeletons in the coffin were preserved in a relatively intact condition.

In November and December 2013, rescue archaeological excavation was carried out at 94 Gyo-dong, Gyeongju-si, Gyeongsangbuk-do province of South Korea before the construction of new driveway by the members of the Silla Cultural Heritage Research Institute (Fig 1A and 1B). The archaeological investigation was conducted under the permission of the Cultural Heritage Administration of Korea (Approval number: 2013–0285). At the district A in the archaeological investigation site, a complete wooden coffin (*mokgwanmyo*) was discovered in a tomb, and human skeletons were found within the coffin (Fig 1C). The size of the coffin was 230 × 80 × 20 cm in length, width and depth respectively. The significance of this tomb was that the human remains were preserved in almost complete form. The surroundings included a marshy area which might contribute to the isolation of the dead body from external destructive environments for a long time. The tomb was also occupied by diverse relics besides the human remains. The analyses of the relics indicated that the tomb was constructed in the Silla period, which also suggest that the human skeletons were from the same period.

Prior to the excavation of the human remains, a public announcement was made based on the Funeral Services Related Act of Korea (Section 2) in order to find possible descendants of the decedent. As the skeletal remains had not been claimed 60 days after the announcement by any possible descendants, the excavation was allowed to proceed with the permission of the Cultural Heritage Administration of Korea (Approval number: 2013–0285). The skeletal remains were moved to the Bio-anthropology Laboratory of Seoul National University Medical College for detailed examination. The specimen and repository numbers were given as GJGD-1 and #321. The repository was involved in the Joseon Dynasty Human Sample Collection of the Department of Anatomy at the Seoul National University College of Medicine (103 Daehak-ro, Chongno-gu, Seoul, South Korea, 03080). The human sample has not been publicly deposited but been allowed to access by other relevant researchers. This study was performed by the permission of the Institutional Review Board of Seoul National University (IRB number: H-0909-049-295).

**Fig 2:**
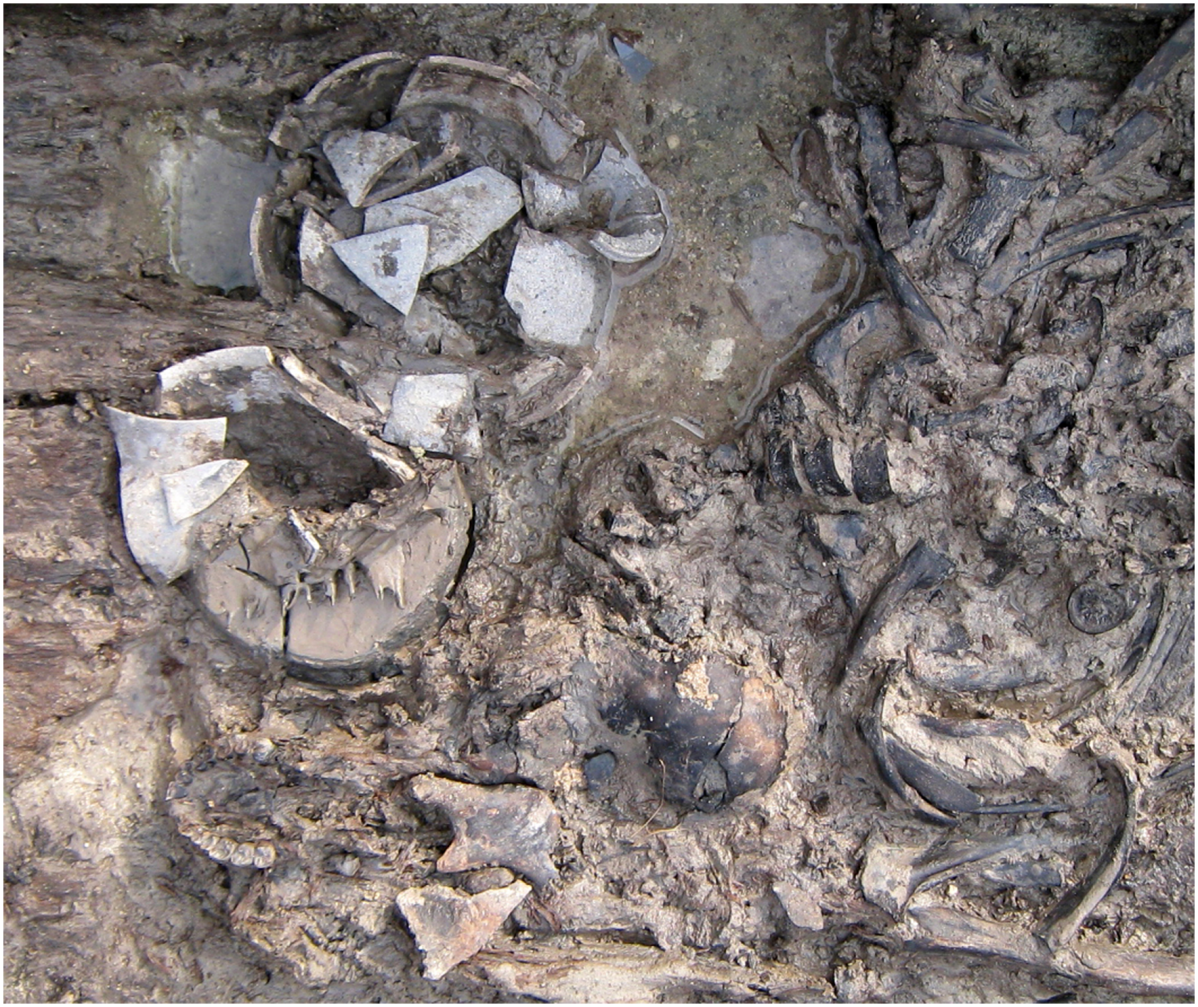
Fragmentary skull portion. The upper body part of the human skeletal remains was magnified from Fig 1C, showing the comingling with other archaeological relics and mud.

Since the osseous fragments were commingled with mud in the archaeological site (Fig 2), all human remains were recovered with the surrounding soil for the transfer to the laboratory. To minimize any contamination, the skeletons in the archaeological site were not allowed to contact with anyone until the removal and transfer from the site to the laboratory had been completed by the excavator (CSO). Every researcher who took part in this study was identified in order to verify cross-contamination during the DNA analysis.

### Physical Anthropological Analysis

The analysis procedure was conducted in accordance with the Vermillion Accord on Human Remains adopted by World Archaeological Congress [5]. Skeletal preparation (cleaning) followed the suggestions described in the text written by White and Folkens [6]. After pre-treatment of the bones, physical anthropological analysis of the specimen was conducted. Stature was determined by measuring the maximum length of the femur following Fujii’s [7] and Pearson’s [8] methods. Sex was determined by morphological analysis using the pelvis and the restored skull [6]. Primary indicators for the sex determination were the greater sciatic notch and pre-auricular sulcus on the hip bone. The auxiliary indicators were morphological features of the skull. Age was estimated by the degenerative changes of the auricular and pubic symphyseal surface on the hip bone [6]. As all the teeth were remained in good condition except for the mandibular left central and lateral incisors, right canine and both 2^nd^ molars, the age at death was estimated by the examination of the length ratios and degenerative change of the pulp cavity of the remaining teeth [9].

### Restoration of the Fragmentary Skull

**Fig 3:**
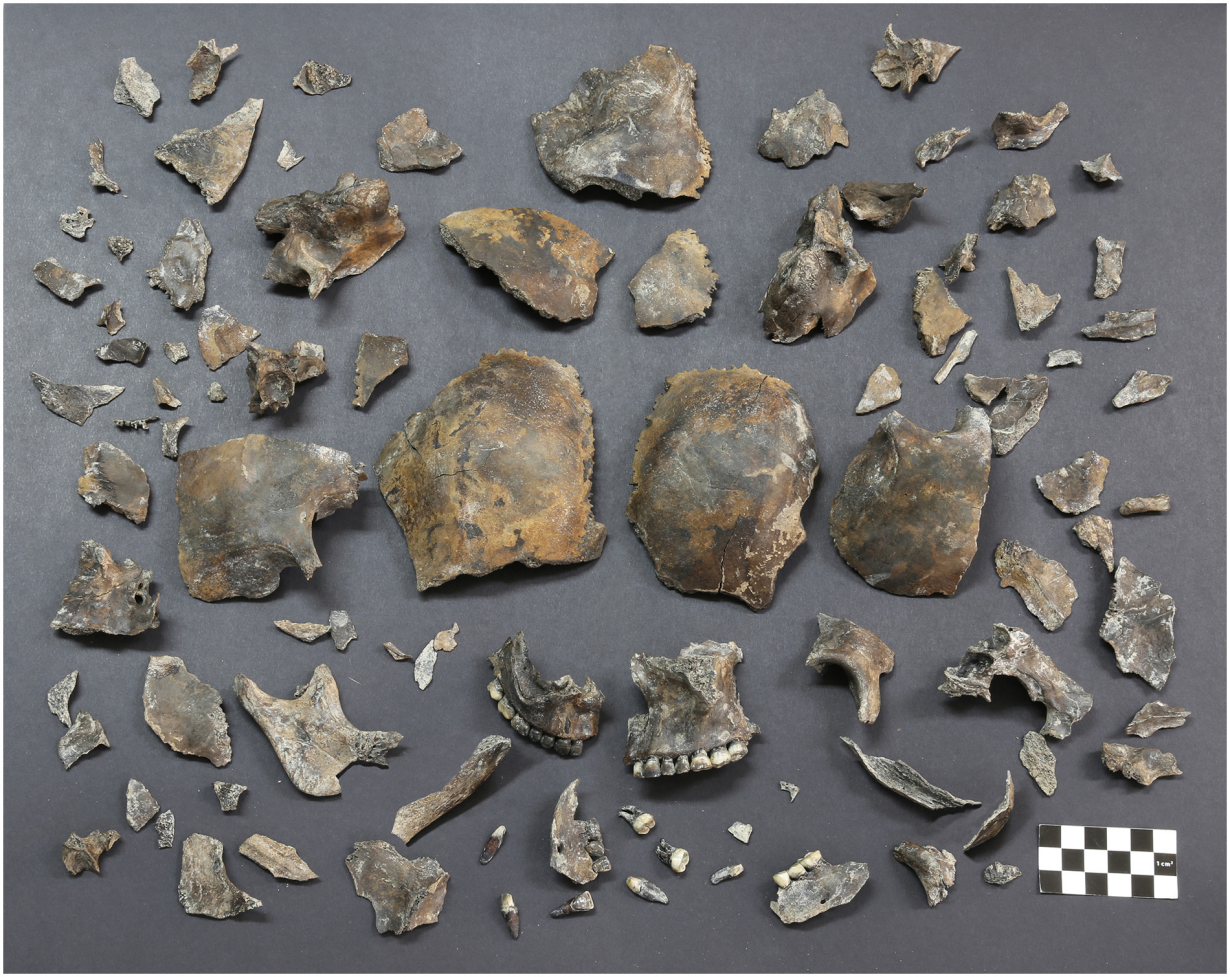
Osseous fragments of the skull after pre-treatment.

Before craniofacial analysis and reconstruction, each osseous piece of the skull was cleaned utilizing lukewarm water with brushes and wooden probes. The washed osseous pieces were dried in room temperature for 48 hours, and prepared for the skull restoration (Fig 3). The restoration procedure was performed in two steps: at first, the virtual restoration with computerized three-dimensional (3D) modelling system (Geomagic^®^ FreeForm^®^ from 3D Systems^®^, USA) to simulate how the fragmentary osseous pieces could be assembled accurately; at second, the real restoration of the skull with actual fragmentary bones referring to the completed virtual restoration.

Each skull fragment were scanned using a cone beam CT (Dentri^™^ from HDXILL^®^, Seoul, Korea) with field of view (FOV) of 16 x 8 cm, a voxel size of 0.20mm and +150 to +200 of Housefield units (Hus). The scanned 3D images of the fragments formatted as Digital Imaging and Communications in Medicine (DICOM) data were converted into stereolithography (STL) formats, and imported into the computerized 3D modelling system for the virtual restoration. Later, the actual fragments of the skull were assembled manually referring to the virtually restored 3D skull model.

### Metric and Paleopathological Analyses

Metric information for the skeleton was measured by digital vernier & spreading caliper (Mitutoyo^®^; Tokyo, Japan) and osteometric board. Available measurements were examined as far as the relevant skeletal portions were observable. In addition, the restored skull and the postcranial skeleton were examined macroscopically to investigate any non-metric traits and pathological changes.

### Craniofacial Reconstruction

The facial morphology and appearance of the individual was depicted by the technique of craniofacial reconstruction (CFR). The CFR using the restored skull was conducted by employing the computerized 3D modeling system. The sex, age and ancestry for the CFR followed the outcomes of physical anthropological analysis. An average facial tissue depths dataset for Korean adults was applied to locate tissue depth gauges at corresponding anatomical landmarks onto the surface of the skull [10]. Each major facial muscle was rebuilt as accurately as possible following anatomical structure of the skull. There are a number of guidelines to predict facial components such as eyes, nose, mouth and ears. The description in detail was stated in the previous studies into Korean CFRs [11,12]. For the final stage, a skin layer was added over the muscle and skull structure referring to the tissue depth gauges by utilizing haptic tools in the computerized modelling system.

### Stable Isotope Analysis

Collagen is extracted from archaeologically obtained bone. Sample was prepared for isotope analysis, following a *modified Longin method*, with the addition of an ultrafiltration step [13]. Briefly, 200mg of powered bone were demineralized in 0.5M HCl solution at 4°C for 16 hour; and then in a pH 3 water, collagen was gelatinized at 75°C for 48 hour. After removing insoluble residues with a 5–8 Ezee^®^ mesh filter, the remaining solution was filtered to remove impurities using 30kDa filters once again [14].

Isotope content and ratio of C and N were determined using a continuous-flow stable isotope ratio mass spectrometer (IsoPrime-EA, Micromass, UK) linked with a CN analyzer (NA Series 2, CE Instruments, Italy). Carbon and nitrogen isotope compositions (*δ*13C and *δ*15N) were calculated as: *δ*(‰) = [(*R*_sample_*/R*_standard_) - 1] × 1000 where *R* is the ratio of ^13^C/^12^C or ^15^N/^14^N; and the standards were the Pee Dee Belemnite (PDB) for carbon and atmospheric (AIR) for nitrogen. Two replicate analyses were carried out for the sample and the average was used for the statistical analysis. Multiple replicate analyses indicated that standard deviations for the *δ*^13^C and *δ*^15^N measurements were *<*0.1‰ and *<*0.2‰, respectively.

### Mitochondrial DNA Analysis

The femur fragment (sample No. 321) was collected with a sterilized knife. This were then exposed to UV irradiation for 20 min, and subsequently immersed in 5.4% (w/v) sodium hypochlorite. When the samples were washed with distilled water and absolute ethanol, they were then air-dried and pulverized to a fine powder using a SPEX 6750 Freezer / Mill (SPEX SamplePrep, Metuchen, NJ) [15]. Bone powder (0.5 g) was incubated in 1 mL of lysis buffer (EDTA 50 mM, pH 8.0; 1 mg /mL of proteinase K; SDS 1%; 0.1 M DTT) at 56°C for 24 h. Total DNA was extracted with an equal volume of phenol / chloroform / isoamyl alcohol (25:24:1), and then was treated with chloroform / isoamyl alcohol (24:1). DNA isolation and purification was performed using a QIAmp PCR purification kit (Qiagen, Hilden, Germany). The purified DNA was eluted in 40 μl of EB buffer (Qiagen) [15].

After quantification was performed by NanoDrop^™^ ND-1000 Spectrophotometer (Thermo Fisher Scientific, MA, USA), 40 ng of the extracted DNA was mixed with the reagent premix containing 1X Ampli*Taq* Gold^®^ 360 Master Mix (Life Technologies, USA) and 10 pmol of each primer (Integrated DNA Technology, USA). PCR conditions used in this study were as follows: pre-denaturation at 94°C for 10 min; 45 cycles of pre-denaturation at 94°C for 10 min; 50 cycles of annealing at 45–56°C for 30 sec; extension at 72°C for 30 sec; final extension at 72°C for 10 min. PCR amplification was performed using a PTC-200 DNA Engine (Bio-Rad Laboratories, Hercules, CA). Primer sets used in this study are summarized in supporting information 1 (S1 Table).

The PCR products were separated on 2.5% agarose gel and then isolated using a Qiagen gel extraction kit (Qiagen, Germany). The sequencing of each amplicon was performed by ABI Prism^®^ 3100 Genetic Analyzer (Applied Biosystems, USA), using ABI Prism^®^ BigDye^™^ Terminator Cycle Sequencing Ready Reaction Kit (Applied Biosystems, USA). Multiple replications of DNA extraction, PCR amplication, cloning and sequencing were performed with an independent laboratory. The obtained DNA sequences were compared with the revised Cambridge Reference Sequence (rCRS; accession number: NC_012920), to identify the sequence differences. The resultant control region mutation motifs were imported into the online-based program *MitoTool* [16] for mtDNA haplogrouping.

In order to minimize any modern DNA contamination of ancient samples, the mtDNA profiles of the researcher (CSO) was determined with the permission of the Institutional Review Board of Seoul National University (IRB number: H-0909-049-295). The researcher was the only person who sampled and carried out mtDNA analysis in this study. The researcher’s mtDNA haplotype was compared with the mtDNA profile from the Silla bone to rule out the possibility of modern DNA contamination. During the mtDNA work, the researcher always wore protection gloves, masks, gowns and head caps. Our mtDNA lab facilities were set up in accordance with the protocol of Hofreiter et al. [17]. The rooms for mtDNA extraction or PCR preparation were physically separated from our main PCR lab. The DNA extraction/PCR preparation rooms were equipped with night UV irradiation, isolated ventilation, and a laminated flow hood.

## Results

### Restoration of the Fragmentary Skull

**Fig 4:**
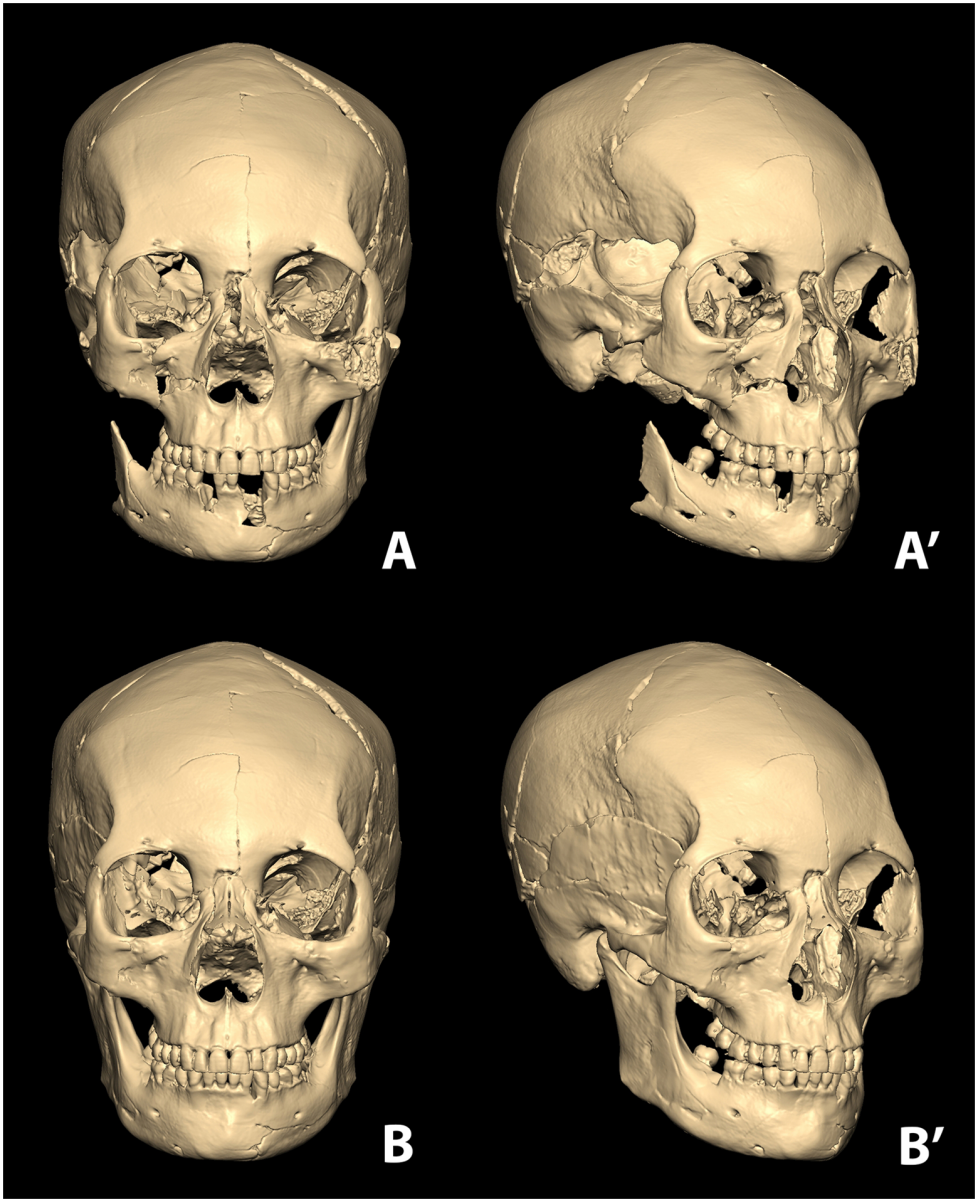
Restored skull in virtual space. The assembled skull from the osseous fragments utilizing computerized 3D modelling program (A and A'). The completed restoration of the skull from damaged/missing parts (B and B').

**Fig 5:**
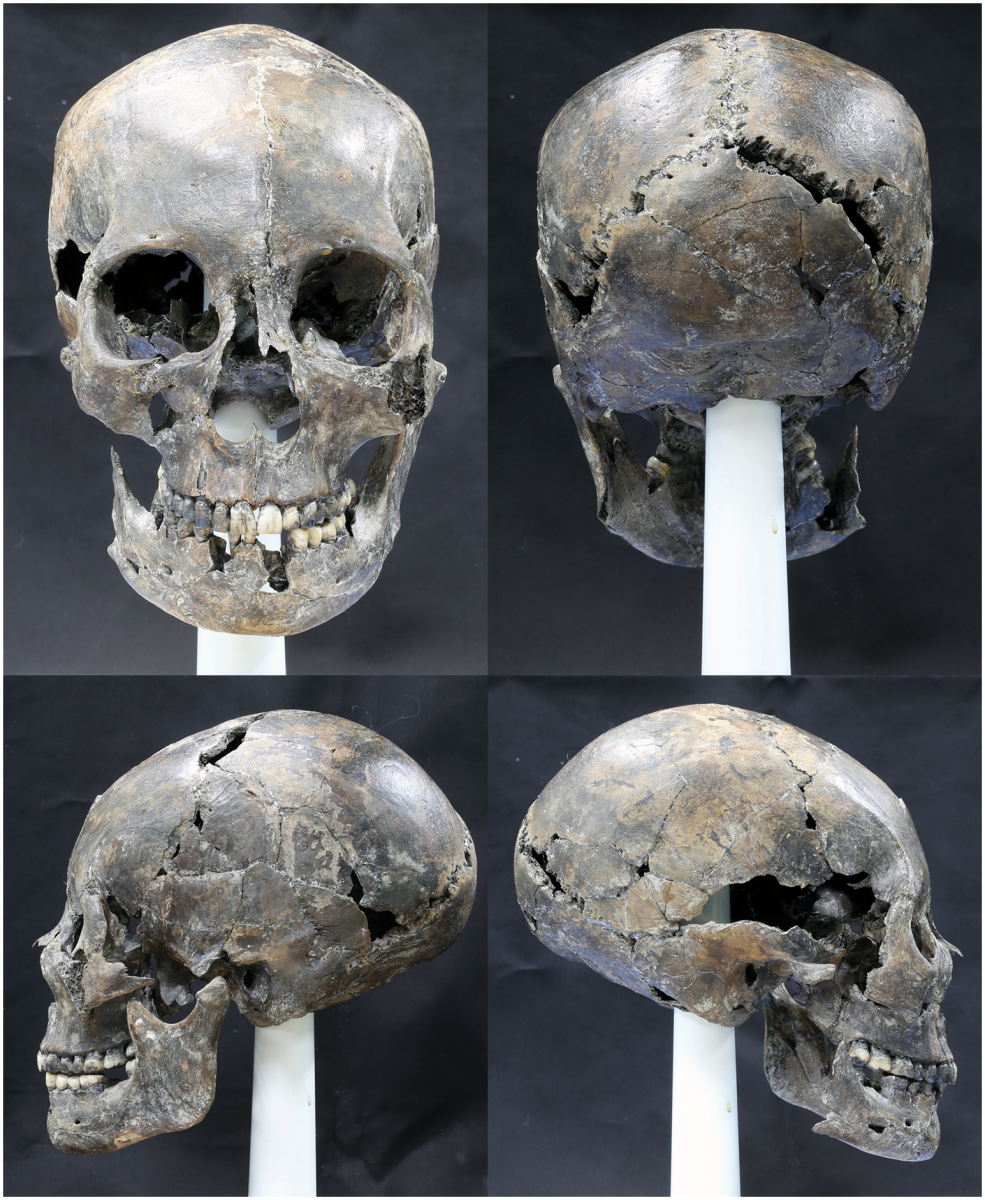
Restored skull in actual space. The assembled skull using actual osseous fragments viewed from four different directions.

With the osseous fragments, the skull was successfully restored using the computerized 3D modeling system (Fig 4A and 4A'). Partially missing parts however were observed on the skull. Although the two enantiomorphs of the human body do not exhibit exact symmetry, the skull could be assumed that both sides may show general symmetry as the restored overall structure did not present any significant asymmetrical morphology. Therefore the missing parts were reconstructed referring to the intact opposite side (Fig 4B and 4B'). Based upon the virtually restored skull model, the actual osseous fragments were easily pieced together (Fig 5).

### Physical Anthropological and Paleopathological analyses

Osteo-metric analysis data collected from the overall skeletons are summarized in supporting information 2 and 3 (S2 and S3 Tables). Based on the pelvic and the skull morphologies, the specimen was determined to be a female (S4 Table, S1 Fig). Additionally, a comb only used by the lady in the era which was found amongst the relics of the tomb suggests that the individual was from a female (S2 Fig). However it was noted that male traits were also observed on the remarkably inclined forehead, everted gonial and developed mental regions on the mandible. DNA-based sex determination was attempted but the analysis did not obtain any results regarding the sex. It was presumed that the nuclear DNA could not be amplified from the ancient specimen of human remains.

By the auricular surface and pubic symphysis on the hip bone [6], the age at death of the specimen was estimated to be 35–39 years and 38.2 ± 10.9 years respectively (S5 Table). According to the Kvaal et al. method using teeth [9], the age at death was estimated to be 35.23 ± 10 years (S6 Table). The advanced degree of teeth attrition suggested that the age at death might be ranged middle-age [6]. Consequently, the age at death of the individual was estimated to be late thirties from the combined results of the four methods.

Stature of the individual was determined by the measurement of maximum length of the femur. Since no regression equation was available for the stature estimation derived from the population group during Silla Kingdom, the result from Fujii’s study which employed modern Japanese subjects [7] was selected to estimate the stature. Following the equation for female (Age (± 4.6) = 2.24 x femur + 61.043), the stature was estimated to be 154.9 ± 4.6 cm. Pearson’s equation for female [8] was also tested because it has been commonly employed to calculate the statures for the human remains from Korea by Korean physical anthropologists [18]. The equation (Age = 1.945 x femur + 72.884) suggested the stature as 154.4 cm.

In the craniometric analysis, the major cranial indices were compared with the corresponding data derived from the subjects of modern Korean adults. The results showed that the skull has longer, narrower and lower cranium with a narrower facial bone and orbits than those from the modern Korean adults groups. The nasal aperture demonstrated an average width in the nasal index [19–21] (Table 1). In terms of appearance, it was assumed that the individual had horizontally long & vertically short head with inclined forehead from lateral view and narrower face from frontal view. The length-breath (cranial) index of the individual can be compared with the results from the ancient and the Joseon Dynasty Korean population groups [22] (Table 2). The index value indicating at 73.5 demonstrated more dolichocephalic than the indices from the two population groups.

**Table 1:** Comparison of the major cranial indices between the modern Korean and the individual of this study.

Study	Index	Male	Female	Results of this study (female)
		Mean ± SD (mm)		(mm)
Hanihara [19]	**Length-breath index**	78.2	Unavailable	73.5
Han et al.[20]	**Length-breath index**	82.7 ± 4.8	84.9 ± 5.2	73.5
	**Length-height index**	81.7 ± 4.6	82.1 ± 3.1	69.7
	**Upper facial index**	54.0 ± 4.2	53.8 ± 3.7	62.7
	**Frontoparietal index**	64.2 ± 3.5	62.7 ± 3.4	65.5
	**Nasal index**	47.1 ± 4.0	50.2 ± 5.1	52.4
Koh et al. [21]	**Orbital index (R)**	77.8 ± 4.6	79.7 ± 5.0	86.8
	**Orbital index (L)**	78.5 ± 4.6	80.9 ± 4.8	90.8

SD, Standard deviation.

**Table 2:** Comparisons of the cranial measurements and indices among female Korean samples from three periods [22].

Measurement	Ancient (4^th^-7^th^ century)	Joseon Dynasty (1392–1910 CE)	Modern
	Mean ± SD (N)	Mean ± SD (N)	Mean ± SD (N)
**Cranial length (mm)**	176.2 ± 8.6 (34)	157.8 ± 5.6 (38)	168.2 ± 7.3 (50)
**Cranial breath (mm)**	136.4 ± 4.9 (37)	137.2 ± 6.7 (38)	138.6 ± 4.8 (50)
**Length-breath index**	77.6 ± 4.9 (28)	82.3 ± 5.4 (35)	82.7 ± 4.6 (50)

N, number of skulls

SD, Standard deviation.

In the visual analysis, no considerable pathological changes were observed on the surface of the skeleton, except for the exostosis lesion on the popliteal surface of the right femur and the inner surface of the right calcaneus.

### Craniofacial Reconstruction

**Fig 6:**
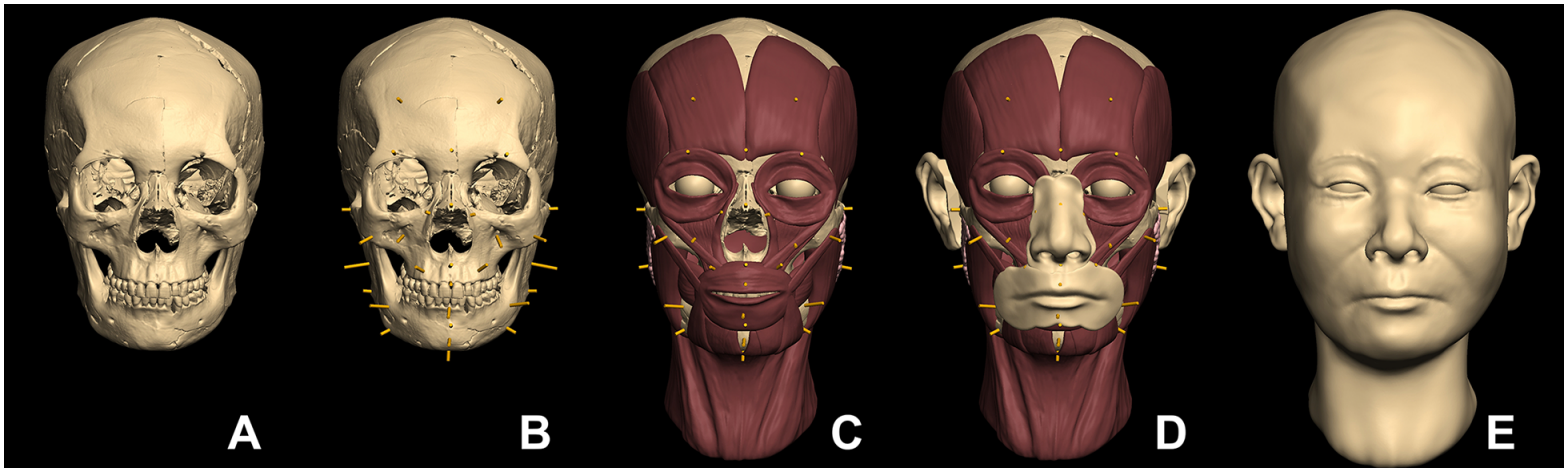
Procedure of the CFR. The restored skull (A). The tissue depth pegs were placed onto the surface of the skull at the corresponding anatomical landmarks (B). Each major facial muscle was rebuilt following anatomical reference guidelines (C). Facial component were placed onto the muscle layer (D). The skin layer was added onto the muscle layer (E).

**Fig 7:**
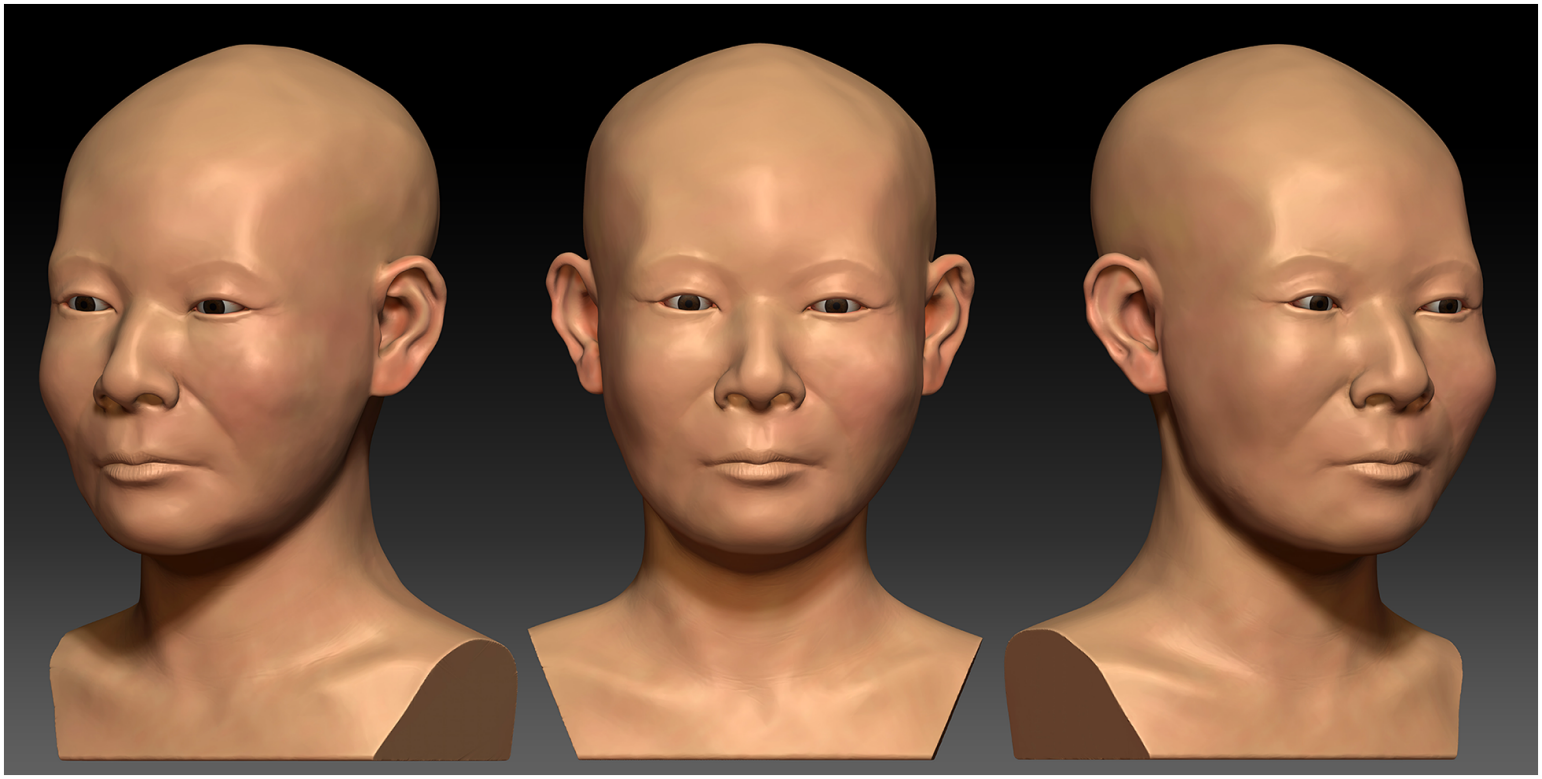
Illustrated CFR. The skin texture was illustrated by computer-assisted graphic software.

The CFR was performed using the restored skull and the combination method [11,12] (Fig 6). In the CFR, the eye fissure was predicted that the right eye fissure has slightly upward exocantus. The eyebrows followed the supra-orbital margin of the skull. The nose was illustrated as showing a bulgy and slightly upturned nasal tip based on the craniometric measurements and analysis. According the palatal width, the individual was expected to have a wider mouth in terms of facial proportions. The skin texture was illustrated onto the CFR utilizing computer-assisted graphic software for the purpose of depicting more realistic facial appearance of the individual (Fig 7).

### Stable Isotope Analysis

**Fig 8:**
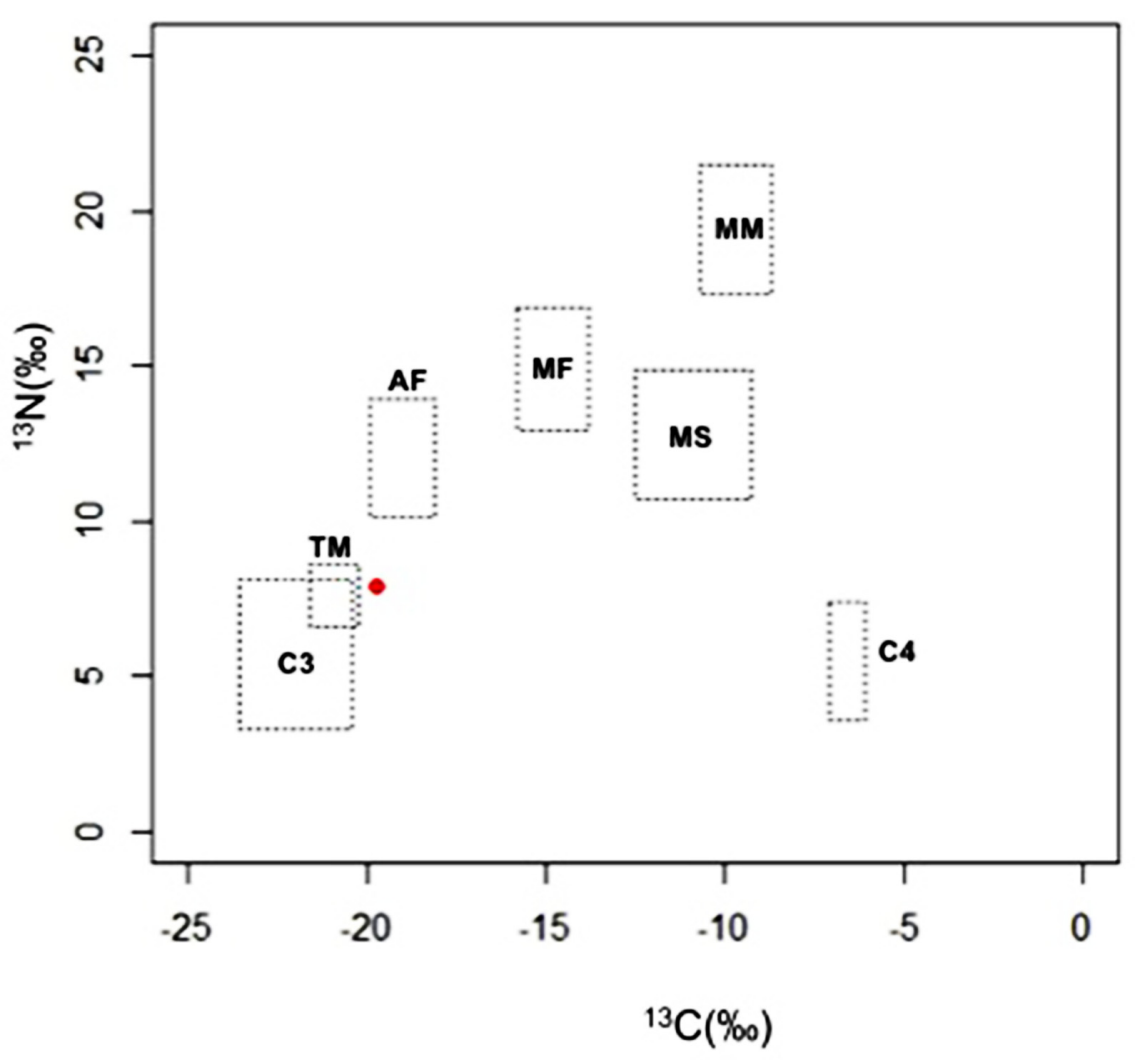
Results from the stable isotope analysis. The red dot presents the mean ± standard deviation of the δ ^13^C and δ ^15^N values from the sample of this study. The squares with dotted lines indicate the mean ± standard deviation of isotope ratios reported for Japanese food groups. C_3_, C_3_ plants; C_4_, C_4_plants; TM, terrestrial mammals; AF, aquatic fishes; MS, marine shellfishes; MF, marine fishes; MM, marine mammals.

The stable isotope result of the Silla individual is summarized in Table 3. The δ13C and δ 15N values, %C and %N, and C:N ratios for individual are described in the same table. For the individual, average of C:N ratio was 3.2, falling within the acceptable range of the analysis. Stable isotope results were -19.72‰ for δ13C and 7.89‰ for δ15N. The δ13C value indicated that Silla people of this study might have consumed more C3- than C4-based foods as the main staples (Fig 8). The δ 15N value for this case was relatively low, even comparing with any of ancient or medieval bones from different countries (Table 4).

**Table 3:** Repeated results of the stable isotope analysis on the human sample.

Test	δ13CPDB (‰)	δ15NAIR (‰)	Total C (%)	Total N (%)	C:N
**Test #1**	-19.80	8.16	46.46	16.85	3.2
**Test #2**	-19.63	7.62	45.93	16.58	3.2
**Average**	-19.72 ± 0.12	7.89 ± 0.38	46.20 ± 0.37	16.72 ± 0.19	3.2

CPDB, The Pee Dee Belemnite (PDB) for carbon; NAIR, The atmospheric (AIR) for nitrogen; C, carbon; N, nitrogen.

**Table 4:** Comparison of the mean stable isotope ratios of adult human from different site and period.

Location		Period	Sex	N	Mean ± SD C(‰)	Mean ± SD N(‰)	Study
**Korea**	**Yeanri**	Gaya (42~562 CE)	F	41	-18.5 ± 0.5	10.1 ± 0.9	Choy K, et al. [14]
			M	28	-18.0 ± 0.6	11.2 ± 0.9	
	**Imdang**	Silla (57 BCE ~ 935 CE)	F	4	-17.6 ± 0.50	9.40 ± 0.90	Shin JY, Lee JJ [23]
			M	5	-17.7 ± 0.60	11.20 ± 0.40	
	**Nukdo**	Late Mumun ~Early Iron (1500 ~ 400 BCE)	F	8	-18.41 ± 0.52	10.90 ± 0.40	Choy KC, Richard MP [24]
			M	5	-18.26 ± 0.23	11.70 ± 1.00	
	**Mungyeong**	Joseon (1392~1910 CE)	F	1	-19.00	11.40	Kang DY, Shin JY [25]
	**Seocheon**	Joseon (1392~1910 CE)	F	2	-20.35 ± 0.35	11.25 ± 0.21	Kang SY, et al. [26]
			M	2	-20.05 ± 0.07	11.80 ± 0.28	
	**Eunpyeong**	Joseon (1392~1910 CE)	F	9	-20.06 ± 0.52	11.77 ± 0.96	Yu JA, et al. [27]
			M	10	-19.70 ± 0.68	11.81 ± 0.58	
	**Sinnae**	Joseon (1392~1910 CE)	F	8	-20.35 ± 0.52	10.43 ± 0.97	
			M	12	-20.10 ± 0.69	11.59 ± 0.58	
	**Unexplained**	Modern* (2010~ CE)	F	4	-19.85 ± 0.44	11.93 ± 0.44	Kang DY, Shin JY [25]
	**Kyeongju**	**Silla** (57 BCE ~ 935 CE)	**F**	**1**	**-19.72 ± 0.12**	**7.89 ± 0.38**	**This study**
**Japan**	**Hitotsubashi**	1657–1683 CE	F	17	-19.30 ± 0.50	10.60 ± 0.80	Tsutaya T, et al. [28]
			M	29	-19.40 ± 0.50	11.30 ± 0.70	
	**Unseiji**	1732 CE	F	1	-19.80	13.9	Nagaoka T, et al. [29]

SD, Standard deviation

N, number of subjects; M, male; F, female.

*hair keratin.

### Mitochondrial DNA Analysis

**Fig 9:**
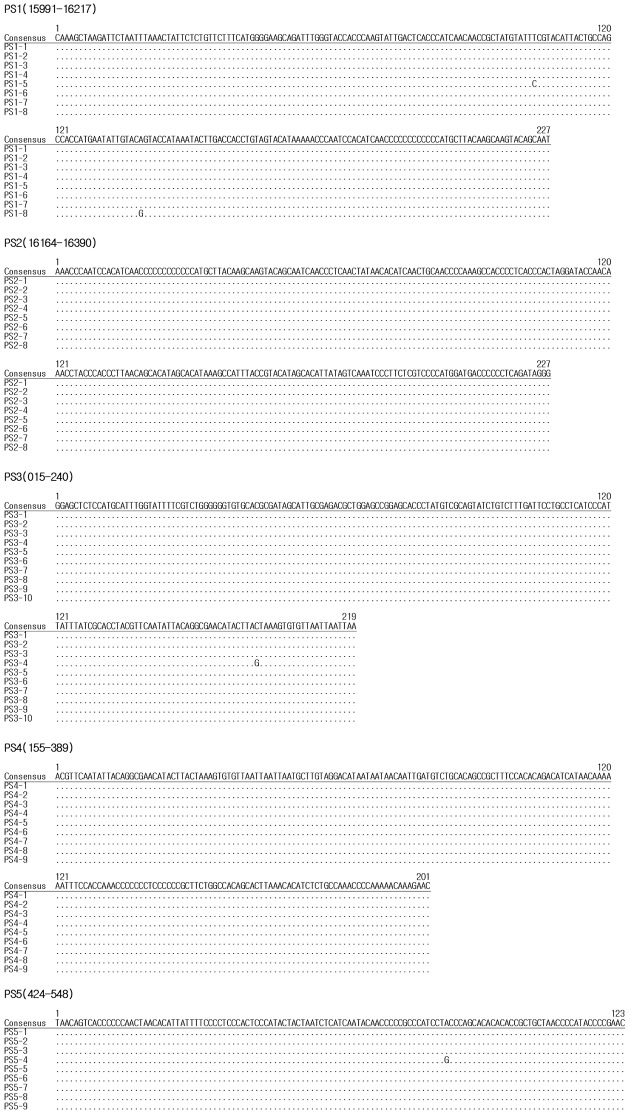
Results from the mtDNA typing analysis. Consensus sequence from nine clones of Silla individual’s mtDNA.

The consensus sequence from nine clones of Silla individual’s mtDNA were obtained. Alignment shows most clone sequences were identical to each other except for only two clones (PS1 B1-1 and PS2 A1-1), showing single nucleotide substitutions. Consensus mtDNA haplotype of the Silla individual was 16220C, 16254G, 16298C, 16362C, 73G, 152C, 249d, 263G, 310.1C (Fig 9). The absence of modern DNA contamination could be confirmed by mtDNA haplotype comparison of hypervariable region from Silla individual and researcher’s samples (Table 5). Since it was confirmed that two mtDNA haplotypes are different from each other, the Silla individual’s mtDNA sequence looks authentic. When haplogroups were assigned by sequence data of the control region, the Silla individual’s haplotype belongs to East Asian haplogroup F1b1a.

**Table 5:** Sequencing analysis result of mtDNA control region.

Subject	Hypervariable region			Haplogroup (mitotool)
	HVI (15991–16390)	HVII (034–369)	HVIII (423–548)	
**#321 (GJGD-1)**	16129A, 16182C, 16183C, 16189C, 16232A, 16249C, 16304C, 16311C, 16344T	73G, 152C, 249del, 263G, 315.1C	514del, 515del	F1b1a
**Researcher 1**	16183C, 16189C, 16220C, 16254G, 16298C, 16362C	73G, 249del, 263G, 315.1C		F3b

## Discussion

Not a few discoveries have been made in relation to the tombs originally constructed for the royal families and nobles during the era of the ancient Korean kingdoms. In those archaeological cases, the human remains were also exhumed from the tombs for the purpose of bio-anthropological studies [14,23]. Although the previous pioneering studies have revealed invaluable information on the biological aspects of the ancient Korean people, our knowledge of the societies of the first millennium is still very incomplete. In this regard, the current study, having employed various investigative tools, provides vivid biological information on one individual from the Silla period.

The restoration of the fragmentary skull was the first important step in this study to acquire physical anthropological information. A number of virtual techniques have been applied for examination of poorly-preserved human specimens. Representatives are reverse engineering, computer-aided design (CAD), and rapid prototyping (RP) techniques [30,31]. However, those tools are rather unnecessarily sophisticated and time-consuming instead, the present study employed a method of virtual skull restoration utilizing a computerized 3D modeling system. By this method, the fragmentary human remains could be assembled very accurately and rapidly. By this technique moreover, missing portions could be repaired efficiently, particularly where an opposite portion of the structure is intact. Of course, the application of what is known as the “mirroring technique” must be carried out with caution where significant asymmetry is observed in the remaining structure.

In the current case, a more reliable craniometric analysis was possible due to the virtually restored skull and the obtained primary craniometric measurements. The cephalic index (CI), at 73.5, suggested a dolichocephalic trait differed from the average or modern Korean adults, who typically demonstrate the brachycephalic or mesochephalic type [19,20]. Reportedly, cranial indices among Koreans changed, from mesochephalic (CI = 77.6 ± 4.9) during the ancient period (4^th^-7^th^ century) to brachychephalic (CI = 82.3 ± 5.4) during the Joseon period (1392–1910 CE) [22,32]. When considering these and the other relevant previous studies [19,20,22,32], the long and narrow cranium and narrower facial shape of the individual in this study should be regarded as an idiosyncratic characteristic of the skull and not a typical feature of East Asians in general or of Koreans in particular. Two hypotheses are possible. The first is that the ancient Koreans living in the region during the Silla period had a narrower cranium than did people living contemporaneously in other regions, the second is that the head of this particular had been artificially narrowed in a cultural rite practiced at that time. The latter however was discounted because, other than the inclined forehead, the cranium did not manifest any typical changes caused by artificial cranial deformation. The former hypothesis seems the more persuasive. One individual however cannot account for any possible variability of the Silla population relative to others of the same era. Thus, further investigation with significantly more numerous skull samples from the period will be necessary.

CFR is a technique used to rebuild a living facial appearance onto a skull in order to recognize or identify an individual. The accuracy of CFR has been of primary importance to maintaining the reliability of its applications to forensic investigation and archaeological research. The most recent study employing quantitative geometric surface comparison found that 75% of CFR surfaces showed less than 2.0 mm of error relative to the corresponding actual faces [11,12,33].

In stable isotope analysis, the carbon isotope ratio δ^13^C shows the proportions of C_3_- (wheat, rice and potatoes) and C_4_-based (maize, millet and other tropical grains) foods in the diets of historical individuals. C_4_-based foods show a higher δ^13^C value than do C_3_-based foods [34]. A previous study determined that the δ^13^C value of C_3_ foods is -25.4 ± 1.6‰ while that of C_4_ grains is -10.0 ± 0.5‰ [35]. The δ^13^C value for the Silla individual examined in the present study was -19.72 ± 0.12‰, indicating that she had consumed a more C_3_-based diet. In fact, this δ^13^C data is similar to the results of studies on Joseon skeletons [25–27], and slightly higher than those for ancient skeletons from Yeanri [14], Imdang [23], and Neukdo [24]. On the other hand, the present *δ* ^15^N value (7.89 ± 0.38‰) is markedly lower than the data on any of the ancient or medieval skeletal remains discovered in South Korea. Judging from a previous study on human bones from a Neolithic settlement in China and modern human hair from England and Germany, it is likely that the present *δ* ^15^N value represents a vegetarian diet [36]. Considering the prevalence of Buddhism among the Silla people of the first millennium, the individual examined in the current study probably practiced strict vegetarianism.

Regarding mtDNA analysis, relevant previous studies already have reported ancient DNA findings on Korean skeletons. Lee et al. [37] presented a genetic characterization of 11 ancient Korean skeletal remains dating to times ranging from the Paleolithic era to the Goryeo Dynasty (918–1392 CE): G3a (Paleolithic), B4f1, D4c, B4b1a1 (Neolithic), D4, D4c1b (Bronze Age), D4e1a (Baekje, 18 BCE-660 CE), and F1a1, D6, A5c, N9a1 (Goryeo). In the current study, however, the Silla individual’s maternal lineage was that of the haplogroup F1b1a. Whereas this is typical of East Asian populations, we must note that it is not the dominant group among modern Koreans [38]. Actually, according to Hong et al. [38], the most common mtDNA sub-haplogroups found in Northeast Asian populations are D4a and D4, followed by A5a and Y1. We admit that our mtDNA results will be truly significant only once a sufficient number of additional cases have been examined in future research. The forthcoming ancient DNA studies on archaeological remains will broaden the information on the genetic traits of ancient Koreans, thereby expanding the knowledge base concerning the migrations of East Asian peoples as well.

## Conclusion

This study, having applied multi-dimensional approaches within an overall bio-anthropological analysis, demonstrates, for the first time, holistic features in the form of biological information on an individual from the ancient Silla period in Korea. The human skeleton examined was estimated to have been that of a female of 155 ± 5 cm height who died in her late 30s. The fragmentary skull was successfully reassembled by reconstructing the damaged or missing portions utilizing computerized 3D modeling software According to a cephalometric analysis, the cephalic index (CI) of the individual was 73.5, which suggests the dolichocephalic trait (long-headedness). This, as compared with similar demographic groups of the era, is unusual. Further, the face of the individual was obtained by craniofacial reconstruction (CFR) technique employing the reconstructed skull and the above-noted computerized 3D modeling. A stable isotope analysis demonstrated that the Silla individual had consumed a more C_3_ (rather than C_4_)-based diet, which, in this particular case, probably was vegetarian. An additional mtDNA analysis showed that the Silla individual’s maternal lineage was that of the haplogroup F1b1a typical of East Asia.

## Supporting Information

S1 Fig Shape of the greater sciatic notch on the right pelvis showing a wide curvature (arrow). (TIF)

S2 Fig A comb used by the female in the era was excavated from the tomb. (TIF)

S1 Table mtDNA primer sequences used in the laboratory 1 and 2. (DOCX)

S2 Table Measurements from craniometric analysis in the restored skull. (DOCX)

S3 Table Measurements from anthropometic analysis in the postcranial skeleton. (DOCX)

S4 Table Results of sex determination from the pelvis and skull. (DOCX)

S5 Table Results of age estimation from the auricular surface and pubic symphysis. (DOCX)

S6 Table Results of age estimation from the teeth. (DOCX)
