# Supplementary figures and images for: Bio-Anthropological Studies on Human Skeletons from the 6th Century Tomb of Ancient Silla Kingdom in South Korea
Source: PLoS One. 2016 Jun 1;11(6):e0156632. doi: 10.1371/journal.pone.0156632 (PMC4889107; doi:10.1371/journal.pone.0156632)

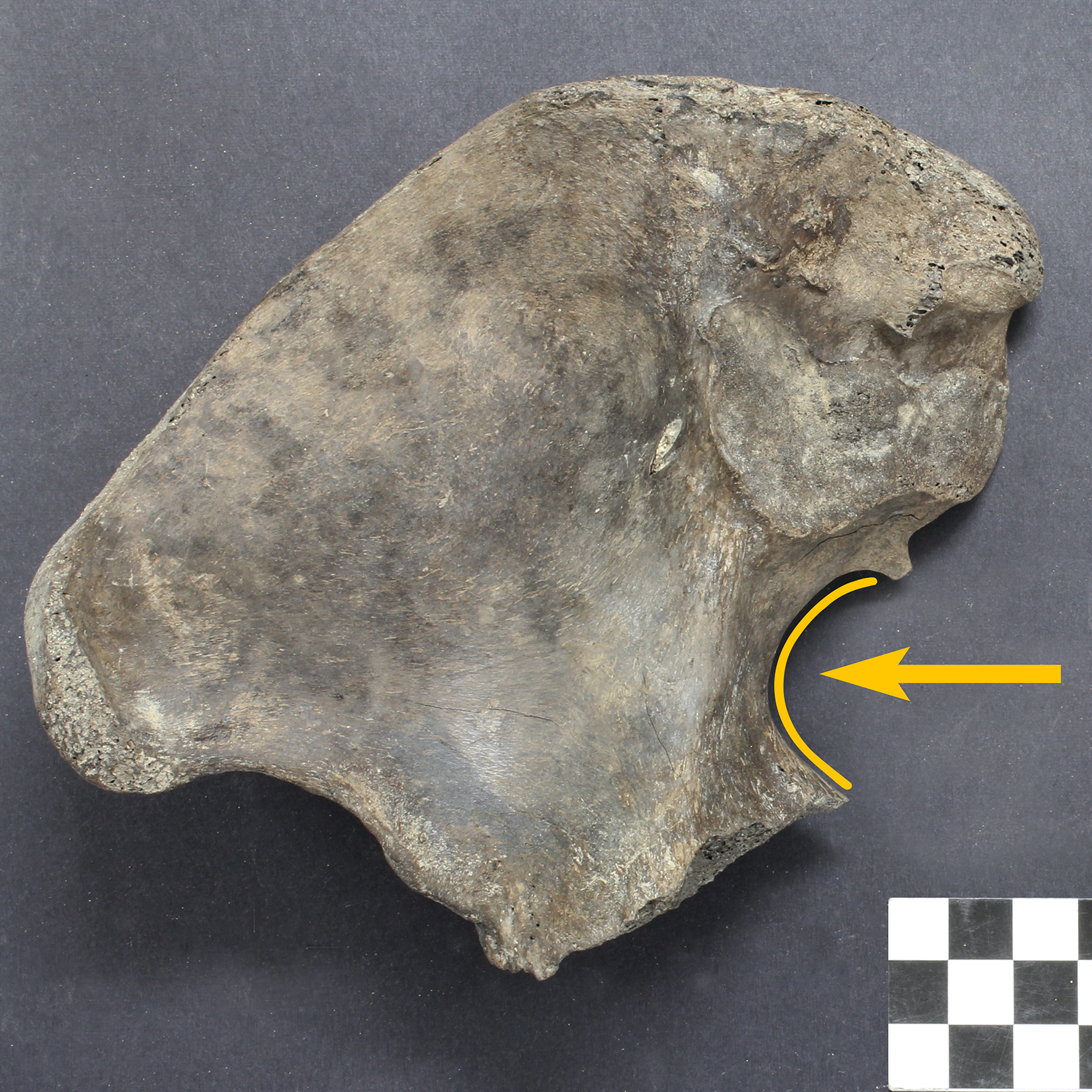

Supplement: S1 Fig — (TIF) [file pone.0156632.s001.tif]

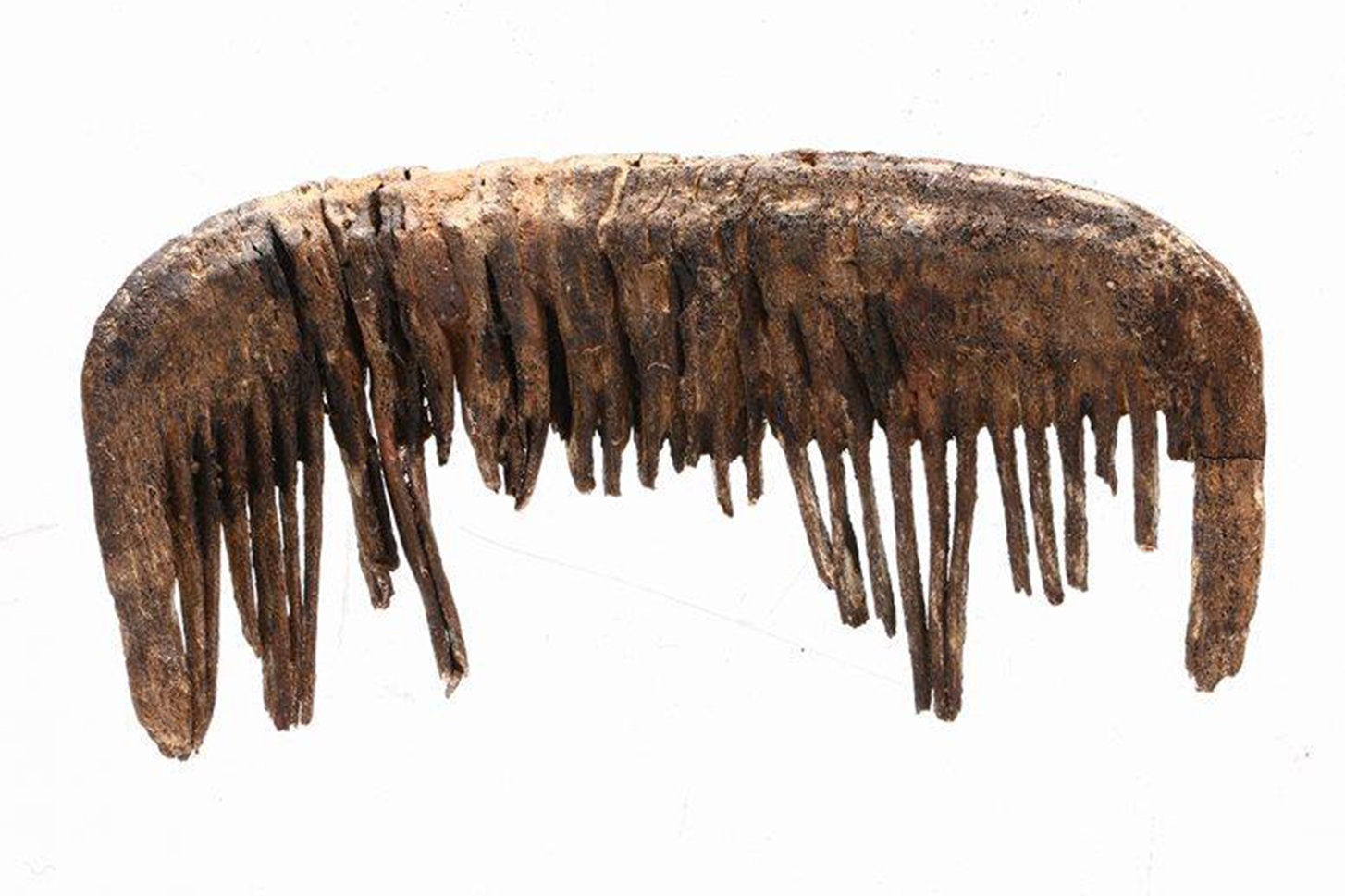

Supplement: S2 Fig — (TIF) [file pone.0156632.s002.tif]
