# Supplementary material for: Bio-Anthropological Studies on Human Skeletons from the 6th Century Tomb of Ancient Silla Kingdom in South Korea
Source: PLoS One. 2016 Jun 1;11(6):e0156632. doi: 10.1371/journal.pone.0156632 (PMC4889107; doi:10.1371/journal.pone.0156632)
Supplement: S1 Table — (DOCX) [file pone.0156632.s003.docx]

**S1 Table. mtDNA primer sequences used in the laboratory 1 and 2.**

| **Lab** | **Region** | **Set** | **Primer** | **Sequence (5’ to 3’)** | **Annealing Temp. (℃)** | **Length (bp)** |
| --- | --- | --- | --- | --- | --- | --- |
| 1 | HVI (15991-16390) | HV1A | F15971 | TTA ACT CCA CCA TTA GCA CC | 56 | 267 |
|  |  |  | R16237 | TGT GTG ATA GTT GAG GGT TG |  |  |
|  |  | HV1B | F16144 | TGA CCA CCT GTA GTA CAT AA | 56 | 267 |
|  |  |  | R16410 | GAG GAT GGT GGT CAA GGG AC |  |  |
|  | HVII (034-369) | HV2A | F015 | CAC CCT ATT AAC CAC TCA CG | 56 | 226 |
|  |  |  | R240 | TAT TAT TAT GTC CTA CAA GCA |  |  |
|  |  | HV2B | F155 | TAT TTA TCG CAC CTA CGT TC | 56 | 235 |
|  |  |  | R389 | CTG GTT AGG CTG GTG TTA GG |  |  |
|  | HVIII (423-548) | HV3 | F403 | TCT TTT GGC GGT ATG CAC TTT | 56 | 167 |
|  |  |  | R569 | GGT GTA TTT GGG GTT TGG TTG |  |  |
| 2 | HVI (15991-16390) | MPS1A | F15989 | CCC AAA GCT AAG ATT CTA AT | 50 | 170 |
|  |  |  | R16158 | TAC TAC AGG TGG TCA AGT AT |  |  |
|  |  | MPS1B | F16112 | CAC CAT GAA TAT TGT ACG GT | 50 | 126 |
|  |  |  | R16237 | TGT GTG ATA GTT GAG GGT TG |  |  |
|  |  | MPS2A | F16190 | CCC CAT GCT TAC AAG CAA GT | 46 | 133 |
|  |  |  | R16322 | TGG CTT TAT GTA CTA TGT AC |  |  |
|  |  | MPS2B | F16268 | CAC TAG GAT ACC AAC AAA CC | 48 | 143 |
|  |  |  | R16410 | GAG GAT GGT GGT CAA GGG AC |  |  |
|  | HVII (034-369) | MPS3A | F34 | GGG AGC TCT CCA TGC ATT TGG TA | 54 | 126 |
|  |  |  | R159 | AAA TAA TAG GAT GAG GCA GGA ATC |  |  |
|  |  | MPS3B | F109 | GCA CCC TAT GTC GCA GTA TCT GTC | 46 | 132 |
|  |  |  | R240 | TAT TAT TAT GTC CTA CAA GCA |  |  |
|  |  | MPS4A | F151 | CTA TTA TTT ATC GCA CCT | 45 | 142 |
|  |  |  | R292 | ATT TTT TGT TAT GAT GTC T |  |  |
|  |  | MPS4B | F220 | TGC TTG TAG GAC ATA ATA AT | 46 | 158 |
|  |  |  | R377 | GTG TTA GGG TTC TTT GTT TT |  |  |
|  | HVIII (423-548) | MVR2 | F403 | TCT TTT GGC GGT ATG CAC TTT | 56 | 167 |
|  |  |  | R569 | GGT GTA TTT GGG GTT TGG TTG |  |  |
