# Supplementary material for: Bio-Anthropological Studies on Human Skeletons from the 6th Century Tomb of Ancient Silla Kingdom in South Korea
Source: PLoS One. 2016 Jun 1;11(6):e0156632. doi: 10.1371/journal.pone.0156632 (PMC4889107; doi:10.1371/journal.pone.0156632)
Supplement: S2 Table — (DOCX) [file pone.0156632.s004.docx]

**S2 Table. Measurements from craniometric analysis in the restored skull.**

| **Part** | **Measurement** | **Value (mm)** |
| --- | --- | --- |
| **Cranium** | Maximum cranial length | 185 |
|  | Maximum cranial breadth | 136 |
|  | Bizygomatic diameter | 112 |
|  | Maximum cranial height | 129 |
|  | Cranial base length | 94 |
|  | Basion-Prosthion length | 95 |
|  | Maxillo-alveolar breadth | 59.2 |
|  | Biauricular breadth | 115.4 |
|  | Upper facial height | 70.2 |
|  | Minimum frontal breadth | 89.1 (biased) |
|  | Upper facial breadth | 107.4 (biased) |
|  | Nasal height | 52.1 |
|  | Nasal breadth | 27.3 |
|  | Orbital breadth (L) | 39.2 |
|  | Orbital breadth (R) | 39.4 |
|  | Orbital height (L) | 35.6 |
|  | Orbital height (R) | 34.2 |
|  | Biorbital breadth | 97.8 |
|  | Interorbital breadth | 24.0 |
|  | Frontal chord | 110.4 |
|  | Parietal chord | 109.7 |
|  | Occipital chord | 100.4 (biased) |
|  | Foramen magnum length | 36.7 |
|  | Foramen magnum breadth | 29.4 |
|  | Mastoid length (L) | 25.1 |
|  | Mastoid length (R) | 28.6 |
| **Mandible** | Chin height | 31.1 |
|  | Body height at mental foramen (L) | 28.6 |
|  | Body height at mental foramen (R) | 29.1 |
|  | Body thickness at mental foramen (L) | 12.0 |
|  | Body thickness at mental foramen (R) | 13.3 |
|  | Minimum ramus breadth (L) | 34.7 |
|  | Maximum ramus height (L) | 59 (biased) |
