# Supplementary material for: Bio-Anthropological Studies on Human Skeletons from the 6th Century Tomb of Ancient Silla Kingdom in South Korea
Source: PLoS One. 2016 Jun 1;11(6):e0156632. doi: 10.1371/journal.pone.0156632 (PMC4889107; doi:10.1371/journal.pone.0156632)
Supplement: S3 Table — (DOCX) [file pone.0156632.s005.docx]

**S3 Table. Measurements from anthropometic analysis in the postcranial skeleton.**

| **Part** | **Left (mm)** | **Right (mm)** |
| --- | --- | --- |
| Clavicle: Maximum Length | 14.2 | - |
| Clavicle: Ant-Post. Diameter at Midshaft | 10.7 | - |
| Clavicle: Sup-Inf. Diameter at Midshaft | 9 | - |
| Humerus: Maximum Length | 300 | - |
| Humerus: Epicondylar Breadth | 56.9 | - |
| Humerus: Vertical Diameter of Head | 41.8 | - |
| Humerus: Maximum Diameter at Midshaft | 18 | 18.4 |
| Humerus: Minimum Diameter at Midshaft | 18 | 18.1 |
| Radius: Maximum Length | 232 | 231 |
| Radius: Anterior-postrior Diameter at Midshaft | 10.2 | 10 |
| Radius: Medial-Lateral Diameter at Midshaft | 13.8 | 14.3 |
| Ulna: Maximum Length | 247 | 250 |
| Ulna: Anterior-Posterior Diameter | 11.2 | 11.4 |
| Ulna; Medial-Lateral Diameter | 13.7 | 12.5 |
| Ulna; Physiological Length | 225 | 229 |
| Ulna; Minimum Circumference | 38 | 38 |
| Sacrum: Anterior Superior Breadth | 114.6 | - |
| Sacrum: Max. Transverse Diameter of Base | 42.5 | - |
| Femur: Maximum Length | - | 419 |
| Femur: Bicondylar Length | - | 411 |
| Femur: Epicondylar Bredth | 76 | 75.2 |
| Femur: Maximum Diameter of the Femur Head | 41.9 | 42.4 |
| Femur: Ant.-Post. Subtrochanteric Diameter | 21.5 | 21.6 |
| Femur: Med.-Lat. Subtrochanteric Diameter | 31.6 | 30.7 |
| Femur: Ant.-Post. Midshaft Diameter | 24.8 | 25.6 |
| Femur: Medial-Lateral Midshaft Diameter | 26.3 | 26 |
| Femur: Midshaft Circumference | 82 | 82 |
| Tibia: Length | 332 | - |
| Tibia: Maximum Proximal Epiphyseal Breadth | 65.6 | - |
| Tibia: Maximum Distal Epiphyseal Breadth | 43.5 | 43.1 |
| Tibia: Max. Diameter at the Nutrient Foramen | 32.2 | 32.7 |
| Tibia: Med.-Lat. Diameter at Nutrient Foramen | 26.6 | 21 |
| Tibia: Circumference at the Nutrient Foramen | 90 | 91 |
| Fibula: Maximum Length | 332 | 333 |
| Fibula: Maximum Diameter at Midshaft | 13.4 | 13.7 |
| Calcaneus: Maximum Length | 78 | 77 |
| Calcaneus: Middle Breadth | 24.9 | 24.2 |
