# Supplementary material for: Bio-Anthropological Studies on Human Skeletons from the 6th Century Tomb of Ancient Silla Kingdom in South Korea
Source: PLoS One. 2016 Jun 1;11(6):e0156632. doi: 10.1371/journal.pone.0156632 (PMC4889107; doi:10.1371/journal.pone.0156632)
Supplement: S4 Table — (DOCX) [file pone.0156632.s006.docx]

**S4 Table. Results of sex determination from the pelvis and skull.**

| **Part** | **Pelvis** | | **Skull** | | | | |
| --- | --- | --- | --- | --- | --- | --- | --- |
| **Structure** | Greater sciatic notch | Pre-auricular sulcus | Nuchal crest | Mastoid process | Supra orbital margin | Glabella | Mental eminence |
| **Score** | 1(Wide) | Present | 1 | 1 | 1 | 2 | 3 |
| **Determination** | Female | | Female | | | | |
