# Supplementary material for: Bio-Anthropological Studies on Human Skeletons from the 6th Century Tomb of Ancient Silla Kingdom in South Korea
Source: PLoS One. 2016 Jun 1;11(6):e0156632. doi: 10.1371/journal.pone.0156632 (PMC4889107; doi:10.1371/journal.pone.0156632)
Supplement: S5 Table — (DOCX) [file pone.0156632.s007.docx]

**S5 Table. Results of age estimation from the auricular surface and pubic symphysis.**

| **Part** | **Auricular surface** | | | | | **Pubic symphysis** |
| --- | --- | --- | --- | --- | --- | --- |
|  | Transverse organization | Porosity | Granularity | Retroauricular activity | Apical activity |  |
| **Score** | No billows  Vague striae | Microporosity | Transition from granula to dense | Slight to moderate | Slight |  |
| **Phase** | Phase IV | | | | | Phase IV |
| **Estimated age range** | 35-39 years | | | | | 38.2 ± 10.9 years |
