# Supplementary material for: Bio-Anthropological Studies on Human Skeletons from the 6th Century Tomb of Ancient Silla Kingdom in South Korea
Source: PLoS One. 2016 Jun 1;11(6):e0156632. doi: 10.1371/journal.pone.0156632 (PMC4889107; doi:10.1371/journal.pone.0156632)
Supplement: S6 Table — (DOCX) [file pone.0156632.s008.docx]

**S6 Table. Results of age estimation from the teeth.**

| **Assessment method** | **element** | **Estimation** |
| --- | --- | --- |
| **Kvaal’s method [9]** | Size of pulp | 31.0 yrs from #11/21  39.0 yrs from #32/42  35.7 yrs from #33/43 |
| **Estimated age range** | 35.23 ± 10 years | |
